# Perceptions of Fibromyalgia Across Career Stages: Survey Results From Internal Medicine Physicians and Medical Students

**DOI:** 10.1002/msc.70253

**Published:** 2026-08-03

**Authors:** Isabel Carulli, Rachel Vanderberg, Kristine Ruppert, Jillian Kyle

**Affiliations:** ^1^ University of Pittsburgh School of Medicine Pittsburgh Pennsylvania USA; ^2^ University of Pittsburgh School of Public Health Pittsburgh Pennsylvania USA

**Keywords:** fibromyalgia, hidden curriculum, medical education

## 1 Background

Despite fibromyalgia's (FM) prevalence and defined diagnostic criteria, providers feel unprepared to care for patients with FM (Arnold et al. 2019; Wolfe et al. 2016). Numerous worldwide studies examining FM perceptions among generalists and specialists have consistently reported provider discomfort in diagnosis and management (Aloush et al. 2021; Arshad and Kong 2007; Byrne et al. 2023; Hadker et al. 2011; Kianmehr et al. 2017; Kumbhare et al. 2018; Perrot et al. 2012; Scott et al. 2023). Multiple societies have published diagnostic criteria; however, several limitations exist including reliance on patient self‐reports, significant symptom overlap with other conditions, and lack of specific objective findings and biomarkers (Benlidayi et al. 2025). A recent systematic review revealed that 51% of physicians view FM as a psychosocial illness and there was substantial management heterogeneity (Agarwal et al. 2024). Clinician discomfort and low knowledge can lead to several negative outcomes, including negative feelings towards patients, strained patient‐provider relationships, mutual frustration, and poor care (Briones‐Vozmediano et al. 2018; Buskila et al. 1997; Byrne et al. 2023; Choy et al. 2010; Colombo et al. 2025; Doebl et al. 2020; Hayes et al. 2010; Ghavidel‐Parsa et al. 2015; Henry and Matthias 2018; Mengshoel et al. 2018; Treufeldt and Burton 2026). Additionally, providers express uncertainty on who should primarily care for patients with FM (Agarwal et al. 2024). While rheumatologists express the least difficulty in FM management, over 80% reported that they should not be the primary FM‐treating physicians (Agarwal et al. 2024; Fernandez‐Avila et al. 2020; Perrot et al. 2012). This results in confusion regarding which specialty should primarily care for patients with FM.

While attending physician attitudes towards FM care are well documented in the literature, few studies focus on medical students' (MS) and internal medicine (IM) residents' perspectives on FM, and those that do are largely qualitative (Amber et al. 2014; Howman et al. 2016; Rice et al. 2017; Vasanthy and Parameswaran Nair 2018). In these studies, trainees reported uncertainty, scepticism, and inexperience similar to those of their attending counterparts. Additionally, trainees can absorb adverse attitudes through the ‘hidden curriculum (HC)’, learning informally by observing preceptors (Lawrence et al. 2018). Prior qualitative work in the United Kingdom and Canada suggested that a lack of formal FM curricula in training may act as an HC, increasing the perception of FM as a low‐priority diagnosis (Shattock et al. 2013; Silverwood et al. 2017). Trainees also acknowledge the hesitancy to question more senior physicians' negative views due to their more extensive experience (Silverwood et al. 2017). This transmission of unfavourable opinions may perpetuate negative stereotypes across successive clinician generations.

This study aimed to assess FM knowledge and attitudes across career stages, investigate U.S. trainees' perceptions of the presence of an HC in FM care, and explore views about which specialty should primarily provide care for patients with FM. To our knowledge, no prior study has quantitatively investigated these topics among trainees or explicitly inquired about HC presence. Recent studies regarding physician and trainee FM attitudes, knowledge and skills are limited. A 2024 systematic review and metanalysis included 21 studies with a single study conducted after 2020 (Agarwal et al. 2024). Given increasing awareness and attention to chronic illnesses such as FM and constantly changing cultural and generational norms, this study provides a timely update regarding trainee and physician FM attitudes (Karimian et al. 2024; Rozenblum and Bates 2013).

## 2 Methods

A cross‐sectional survey was conducted at a large academic medical centre from April through June 2024. Participants included community and academic IM attendings, IM residents, and third‐ and fourth‐year MS. All eligible individuals were invited to participate via electronic mail outreach with a link to an online survey. Survey data were collected and managed using REDCap electronic data capture tools (Harris et al. 2009, 2019). Surveys remained anonymous and distinctive by using unique identifiers. Only study authors had access to the data which was housed on the REDCap platform on a secure institutional online server. The survey timing in late spring ensured that all MS patients and residents had completed at least one outpatient medicine rotation. Prospective participants were incentivised by entry into a random drawing for a gift card on survey completion.

Participants completed four surveys: (1) perceived FM knowledge and which specialty should primarily provide FM care, using five‐point Likert scales (1 = none/nothing to 5 = a great deal and 1 = strongly disagree to 5 = strongly agree); (2) an adapted validated tool, the Difficult Doctor‐Patient Relationship Questionnaire (DDPRQ‐10), to assess personal attitudes towards patients with FM, using a six‐point Likert scale (1 = not at all to 6 = a great deal); (3) trainee perceptions of a HC in FM care, using a five‐point Likert scale (1 = strongly disagree to 5 = strongly agree), as well as their perceptions of preceptor attitudes towards patients with FM, using an adapted DDPRQ‐10; and (4) a demographic survey (Hahn 2001). All surveys were developed de novo for this study except for the adapted DDPRQ‐10. For the adapted DDPRQ‐10, original items were not altered other than to explicitly state that questions referred to FM, and to define whether the question referred to the participant's opinion or the trainee's perceived opinion of their preceptors. The DDPRQ‐10 has previously been applied to FM in other studies (Aloush et al. 2021; Homma et al. 2016). Before implementation, all surveys were reviewed and iteratively revised with the help of three experienced physicians in FM care (Supporting Information S1: Appendix 1). Surveys were pilot tested among six volunteers including IM physicians, residents, and MS with variable FM experience.

Descriptive characteristics were calculated using means with standard deviations for continuous variables and counts with percentages for categorical variables. To examine the differences in knowledge and attitudes between patients with MS, residents and attendings, unadjusted generalised linear models (GLM) were used (SAS Institute, 2023). To determine differences between the three groups, Tukey's correction for multiple comparisons was applied. *p* values < 0.05 are considered significant. All analyses were performed using SAS 9.4 (Cary, NC). This study was approved as exempt by the University of Pittsburgh Institutional Review Board on 5 April 2024 (STUDY 23070092). Funding was provided by the Competitive Research Fund of Shadyside Hospital Foundation; the foundation had no role at any stage of study development, implementation, or data analysis.

## 3 Results and Discussion

**FIGURE 1:**
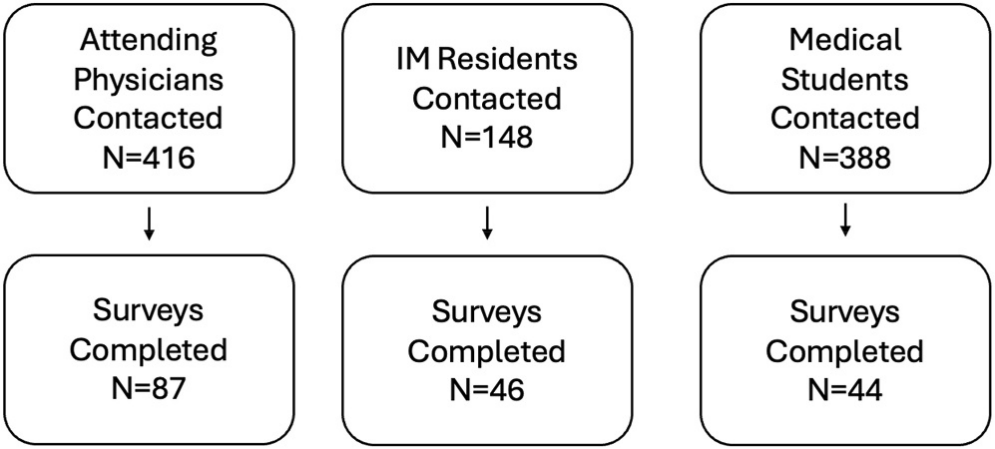
Participant recruitment.

Eleven percent of MS (44/388), 31% of residents (46/148), and 21% of attendings (87/416) completed the survey (Figure 1). Table 1 details participant demographic information. Table 2 details perceived knowledge and attitudes about FM. All groups rated their knowledge as inadequate; however, attendings reported significantly higher perceived knowledge of symptoms, diagnosis, and treatment than residents, and significantly higher symptom and treatment knowledge than MS (all *p* < 0.05). All groups felt neutral regarding whether FM is a defined medical disorder with clear diagnostic criteria, with MS more likely to disagree with this statement (*p* < 0.05). All groups felt neutral on whether primary care physicians should be primarily responsible for FM management and disagreed that specialists should take primary responsibility. MS expressed more neutral feelings than their attending counterparts on whether specialties other than primary care should take a primary role in patient management (*p* < 0.05).

**TABLE 1:** Participant demographics.

	Attendings (87)		Residents (46)		Medical students (44)	
	*n* (%)		*n* (%)		*n* (%)	
Age	< 30	1 (1)	< 25	0	< 25	2 (5)
	30–40	28 (32)	25–30	29 (63)	≥ 25	26 (59)
	41–50	18 (21)	31–35	9 (20)	UA	16 (36)
	51–60	13 (15)	≥ 35	2 (4)		
	61–70	8 (9)	UA	6 (13)		
	> 71	2 (2)				
	UA	17 (20)				
Gender						
Female	41 (47)		25 (54)		18 (41)	
Male	29 (33)		15 (33)		8 (18)	
Prefer not to say	0		0		2 (5)	
UA^a^	17 (20)		6 (13)		16 (36)	
Race/ethnic group						
Alaska Native/American Indian	0		0		0	
Asian	12 (14)		9 (20)		10 (23)	
Black	0		0		3 (7)	
Hispanic	1 (1)		3 (7)		2 (5)	
Native Hawaiian/Pacific Islander	0		0		0	
White	57 (66)		23 (50)		13 (30)	
Prefer not to say	2 (2)		7 (15)		3 (7)	
UA	15 (17)		4 (9)		13 (30)	
Patients in typical week with FM						
0–5	56 (64)		—		—	
6–10	12 (14)		—		—	
> 10	2 (2)		—		—	
UA	17 (20)		—		—	
Non‐clinical experience with FM						
Family or friend	4 (5)		3 (7)		2 (5)	
Personal diagnosis	0		0		0	
Prefer not to say	2 (2)		0		1 (2)	
UA	81		43 (93)		41 (93)	
Residency year						
PGY1	—		17 (37)		—	
PGY2	—		10 (22)		—	
PGY3	—		13 (28)		—	
UA	—		6 (13)		—	
FM didactic experience (in hours)						
0	—		—		9 (20)	
1–2	—		—		12 (27)	
3–5	—		—		5 (11)	
> 5	—		—		2 (5)	
UA	—		—		16 (36)	

*Note:* Given the small number of participants, all sections are number of participants, not percentages.

Abbreviations: FM, fibromyalgia; UA, unanswered.

^a^ A non‐binary option was provided but not chosen by participants.

**TABLE 2:** Fibromyalgia (FM) perceived knowledge and attitudes among medical students, residents and attendings.

Question	Medical students (MS)	Residents (R)	Attendings (A)	Overall *F*‐statistic	*p* value^a^ (differences)
	Mean (SD)	Mean (SD)	Mean (SD)		
FM is a primarily psychological disorder^b^	2.55 (0.87)	2.54 (0.87)	2.52 (0.97)	0.98	—
FM is a defined disorder with clear diagnostic criteria^b^	3.14 (1.03)	3.76 (1.04)	3.60 (1.16)	0.06	—
Primary care physicians are primarily responsible for management^b^	3.72 (0.80)	3.66 (0.85)	3.99 (0.75)	0.07	—
Rheumatologists are primarily responsible for management^b^	3.17 (0.80)	2.59 (0.92)	2.38 (1.05)	< 0.01	< 0.001 (MS vs. A)
Neurologists are primarily responsible for management^b^	2.62 (0.68)	2.10 (0.58)	1.81 (0.80)	< 0.01	< 0.001 (MS vs. A)
Physiatrists are primarily responsible for management^b^	3.21 (1.01)	2.51 (0.78)	2.70 (1.04)	0.01	0.01 (MS vs. R)
					0.04 (MS vs. A)
Psychiatrists are primarily responsible for management^b^	2.83 (0.80)	2.22 (0.69)	2.09 (0.96)	< 0.01	1.1 (MS vs. R)
					0.003 (MS vs. A)
How much do you feel you know about the signs and symptoms of FM?^c^	3.70 (0.72)	3.52 (0.71)	3.96 (0.67)	< 0.01	1.1 (MS vs. A)
					< 0.001 (R vs. A)
How much do you feel you know about how to diagnose FM?^c^	3.34 (0.87)	3.24 (0.83)	3.69 (0.78)	< 0.01	0.01 (R vs. A)
How much do you feel you know about how to treat FM?^c^	3.35 (0.87)	3.29 (0.86)	3.82 (0.81)	< 0.01	1.1 (MS vs. A)
					0.003 (R vs. A)

Abbreviation: SD, standard deviation.

^a^ Least Square Means of Effect Test determining which groups showed significant differences.

^b^ Likert Scale for attitude questions: 1 = none, 2 = a little, 3 = some, 4 = much, 5 = a great deal.

^c^ Likert Scale for knowledge questions: 1 = nothing; 2 = very little; 3 = a little; 4 = a moderate amount; 5 = a great deal.

Table 3 demonstrates the results of the adapted DDPRQ‐10. All groups rated patients with FM as a little to somewhat frustrating, particularly regarding vague complaints, and felt only a little to somewhat at ease with these patients. All groups felt that patients were somewhat to moderately time‐consuming. They largely disagreed that patients with FM were manipulative, difficult to communicate with, and did not dread their return. Patients with MS were significantly less likely to find them frustrating, difficult to communicate with or evoking dread. They were also less likely to find patients manipulative or time‐consuming when compared with attending physicians (*p* < 0.05). When asked about their attending preceptors, neither trainee group clearly perceived the presence of an HC; both trainee groups had positive perceptions of attending attitudes about patients with FM.

**TABLE 3:** Adapted Difficult Doctor–Patient Relationship Questionnaire‐10 (DDPRQ‐10) results for all groups, trainee DDPRQ‐10 perceptions of attendings, and perception of a hidden curriculum around fibromyalgia (FM).

Question	Medical students (MS)	Residents (R)	Attendings (A)	Overall *F*‐statistic	*p* value^a^ (differences)
	Mean (SD)	Mean (SD)	Mean (SD)		
How much are you looking forward to seeing patients with FM^b^	2.90 (0.94)	2.12 (1.21)	2.54 (1.12)	0.02	0.01 (MS vs. R)
How frustrating do you find patients with FM^b^	2.28 (0.99)	3.49 (1.23)	3.30 (1.18)	< 0.01	0.001 (MS vs. R)
					< 0.001 (MS vs. A)
How manipulative do you find patients with FM^b^	1.41 (0.73)	1.95 (1.18)	2.25 (1.51)	0.03	0.01 (MS vs. A)
How frustrated are you with FM patients' vague complaints^b^	2.45 (1.12)	3.15 (1.26)	3.09 (1.30)	0.04	—
How self‐destructive do you find patients with FM^b^	1.59 (0.95)	2.02 (1.13)	2.21 (1.39)	0.08	—
How much do you find yourself secretly hoping patients with FM will not return^b^	1.48 (0.78)	2.51 (1.29)	2.26 (1.34)	< 0.01	0.002 (MS vs. R)
					0.01 (MS vs. A)
How much do you feel at ease with patients with FM^b^	3.38 (0.94)	2.54 (0.95)	3.34 (1.15)	< 0.01	0.004 (MS vs. R)
					< 0.001 (R vs. A)
How time‐consuming do you find caring for patients with FM^b^	3.45 (1.18)	4.07 (1.08)	4.09 (1.28)	0.04	0.04 (MS vs. A)
How enthusiastic do you feel about caring for FM patients^b^	2.83 (1.23)	2.41 (1.14)	2.47 (1.11)	0.27	—
How difficult is it for you to communicate with FM patients^b^	1.83 (0.89)	2.88 (1.21)	2.54 (1.38)	< 0.01	0.002 (MS vs. R)
How much does your preceptor look forward to seeing patients with FM^b^	3.50 (1.79)	2.81 (1.47)	—	0.33	—
How frustrating does your preceptor find patients with FM^b^	2.72 (1.18)	1.94 (0.68)	—	0.03	—
How manipulative does your preceptor find patients with FM^b^	1.67 (1.08)	1.63 (0.81)	—	0.90	—
How frustrated is your preceptor by patients with FM's vague complaints^b^	2.28 (1.02)	2.13 (1.09)	—	0.68	—
How self‐destructive does your preceptor find patients with FM^b^	1.56 (1.15)	1.56 (0.81)	—	0.98	—
How much does your preceptor secretly hope patients with FM will not return^b^	1.5 (0.62)	1.63 (0.81)	—	0.61	—
How at ease does your preceptor feel with patients with FM^b^	3.67 (1.53)	3.19 (1.28)	—	0.33	—
How enthusiastic is your preceptor about caring for patients with FM^b^	3.17 (1.62)	2.88 (1.31)	—	0.65	—
How difficult is it for your preceptor to communicate with patients with FM^b^	1.89 (0.83)	2.19 (1.17)	—	0.57	—
I feel there is a hidden curriculum that patients with FM are difficult to manage^c^	2.76 (0.99)	3.19 (1.22)	—	0.39	—

Abbreviation: SD, standard deviation.

^a^ Least Square Means of Effect Test determining which groups showed significant differences.

^b^ Scale for DDPRQ‐10 questions: 1 = not at all, 2 = a little, 3 = somewhat, 4 = moderately, 5 = much, 6 = a great deal.

^c^ Likert Scale for hidden curriculum question: 1 = strongly disagree, 2 = disagree, 3 = neutral, 4 = agree, 5 = strongly agree.

Our study is congruent with prior findings of both negative and ambivalent FM attitudes among attendings and expands our understanding of trainees' views (Amber et al. 2014; Howman et al. 2016; Rice et al. 2017; Vasanthy and Parameswaran Nair 2018). Encouragingly, and in contrast to prior studies (Shattock et al. 2013; Silverwood et al. 2017), both MS and residents viewed attendings as having positive views of patients with FM, did not definitively perceive a HC (although subtle implicit biases may still exist), and had more positive views themselves of patients with FM. Prior work has demonstrated that patients with MS are more likely to favour a physiological mechanism to FM than their attending counterparts, who were more likely to question or attribute symptoms to a psychological aetiology (Amber et al. 2014). The reasons for more favourable attitudes among trainees are unclear but may include a generational shift towards more empathic, patient‐centred care for chronic illnesses given increased attention to these conditions and patients' documentation of their lived experiences on social media (Bell et al. 2024; Karimian et al. 2024; Rozenblum and Bates 2013). Additionally, medical school and residency programs now increasingly recognise implicit bias in training and have increased education to improve attitudes towards traditionally marginalised groups (Geller and Watkins 2018; Ruben and Saks 2020; Walker et al. 2025). The disconnect between attending providers' more negative views and trainees' positive perceptions of their mentors may suggest that attendings mask their ambivalence towards these patients well or that trainees are less attuned to subtle biases of their preceptors. While current trainees may exhibit decreased negative attitudes in the future compared to their current attendings, physicians may develop negative attitudes towards FM with exposure and time, regardless of their educational experience.

Despite more positive attitudes among trainees in this study, knowledge of FM remains low across groups, with no consensus on whether FM is a clearly defined disorder. Attending participants reported low numbers of FM patients in clinical practice, whereas students reported limited exposure to FM education, which may in part explain the low perceived knowledge. In previous studies, physician‐reported insufficient knowledge was associated with frustration and negative attitudes in caring for patients with FM (Hayes et al. 2010). Repeated exposure of trainees to patients with FM could improve knowledge and subsequent attitudes. Prior work has shown that trainees desire increased instruction in this area, and that having positive repeated encounters with patients with complex syndromes improved attitudes (Howman et al. 2016; Silverwood et al. 2017).

This study supports prior findings of physician uncertainty regarding who should primarily manage FM (Agarwal et al. 2024; Fernandez‐Avila et al. 2020; Perrot et al. 2012) and expands this uncertainty to trainees. Previous work demonstrated that rheumatologists are most comfortable in caring for patients with FM, but do not seek this responsibility (Agarwal et al. 2024; Fernandez‐Avila et al. 2020; Perrot et al. 2012). Generalist physicians and other specialties are uncomfortable in FM management. Unfortunately, this leaves patients in a quandary without a clearly designated primary provider for FM care. A potential solution is the development of FM specialty clinics. Previous work has shown that FM clinics result in higher levels of patient symptom improvement, satisfaction, and sense of belonging (Giusti et al. 2017; Häuser et al. 2009; Pressimone et al. 2024, 2026). Although FM specialty clinics could be a potential solution, it is likely that this care delivery model may not be feasible in under resourced areas or health systems. Further research into FM care models is needed to fully elucidate the most appropriate care delivery model for high quality, evidence‐based FM care.

There are notable limitations to this study. The adapted DDPRQ‐10 has not been specifically validated. This version's minor language edits are unlikely to change the scale's validity, however, formal validation has not been completed. Another limitation is the low response rate; however, the data resembles that of prior studies on FM attitudes (Briones‐Vozmediano et al. 2018; Buskila et al. 1997; Doebl et al. 2020; Hayes et al. 2010; Henry and Matthias 2018). The low sample size may reflect the impersonal nature of electronic mail recruiting, bias around FM diagnostic legitimacy, or perceived lack of benefit in completing the survey. It is possible that only those with stronger opinions felt compelled to respond. If trainees with overall more positive views of their training and attendings respond, they may perceive less HC presence than those with more negative experiences. Additional recruitment strategies such as in‐person recruiting and dedicated completion time may increase the response rate and elicit more varied perspectives. An additional limitation is both the low participant response to demographic questions as well as the non‐representative sample in terms of ethnicity and gender. This may be due to the nature of the topic, survey length, and demographic question placement at the survey's end. The non‐representative sample and the study's single institution design may not be generalisable to more diverse centres, practice types, or different institutional cultures. Increasing the number of institutions may provide a more representative demographic sample and may gather perspectives from centres with variable FM education and/or FM patient exposure.

Next steps include expanding the study to multiple institutions to better understand the scope of FM attitudes in trainees. Greater collaborative agreement among specialties about which provider should offer primary FM care and increased education at all levels to enhance FM knowledge, skills and attitudes, may improve FM‐related attitudes, as well as satisfaction among patients and physicians.

## Author Contributions

J.K. and R.V. conceived the original idea. I.C., J.K., and R.V. designed the surveys, recruited participants, and administered the surveys. K.R. completed the statistical analysis. All authors were critically involved in the interpretation of the results. I.C. wrote the manuscript with support from J.K., K.R., and R.V. All authors have contributed to the review and revision of the manuscript.

## Funding

This study was funded by the Thomas H. Nimick Jr. Competitive Research Fund and the National Institutes of Health (Grant UL1TR001857).

## Conflicts of Interest

The authors declare no conflicts of interest.

## Supporting information


Supporting Information S1

## Data Availability

The datasets generated during and/or analysed during the current study are available from the corresponding author on reasonable request.

## References

[msc70253-bib-0001] Agarwal, A. , P. C. Emary , L. Gallo , et al. 2024. “Physicians' Knowledge, Attitudes, and Practices Regarding Fibromyalgia: A Systematic Review and Meta‐Analysis of Cross‐Sectional Studies.” Medicine 103, no. 31: e39109. 10.1097/MD.0000000000039109. 39093781 PMC11296454

[msc70253-bib-0002] Aloush, V. , D. Niv , J. N. Ablin , I. Yaish , O. Elkayam , and O. Elkana . 2021. “Good Pain, Bad Pain: Illness Perception and Physician Attitudes Towards Rheumatoid Arthritis and Fibromyalgia Patients.” Clinical & Experimental Rheumatology 39, no. 3: 54–60. 10.55563/clinexprheumatol/u1nbxz. 33338002

[msc70253-bib-0003] Amber, K. , L. Brooks , J. Chee , and T. S. Ference . 2014. “Assessing the Perceptions of Fibromyalgia Syndrome in the United States Among Academic Physicians and Medical Students: Where Are We and Where Are We Headed?” Journal of Musculoskeletal Pain 22, no. 1: 13–19. 10.3109/10582452.2014.883024.

[msc70253-bib-0004] Arnold, L. M. , R. M. Bennett , L. J. Crofford , et al. 2019. “AAPT Diagnostic Criteria for Fibromyalgia.” Journal of Pain 20, no. 6: 611–628. 10.1016/j.jpain.2018.10.008. 30453109

[msc70253-bib-0005] Arshad, A. , and K. O. Kong . 2007. “Awareness and Perceptions of Fibromyalgia Syndrome: A Survey of Malaysian and Singaporean Rheumatologists.” Singapore Medical Journal 48, no. 1: 25–30. https://www.sma.org.sg/smj/4801/4801a3.pdf. 17245512

[msc70253-bib-0006] Bell, L. , B. Fordham , S. Mumtaz , et al. 2024. “Using Natural Language Processing and Social Media Data to Understand the Lived Experience of People With Fibromyalgia.” Healthcare 12, no. 24: 2511. 10.3390/healthcare12242511. 39765938 PMC11728136

[msc70253-bib-0007] Benlidayi, I. C. , C. Ornek , V. Deniz , and A. Sariyildiz . 2025. “Reevaluating Fibromyalgia Diagnosis: A Proposal to Integrate Deep Tendon Reflex Responses Into Current Criteria.” Rheumatology International 45, no. 4: 84. 10.1007/s00296-025-05846-y. 40172661 PMC11965154

[msc70253-bib-0008] Briones‐Vozmediano, E. , A. Öhman , I. Goicolea , and C. Vives‐Cases . 2018. “‘The Complaining Women’: Health Professionals’ Perceptions on Patients With Fibromyalgia in Spain.” Disability & Rehabilitation 40, no. 14: 1679–1685. 10.1080/09638288.2017.1306759. 28385050

[msc70253-bib-0009] Buskila, D. , L. Neumann , D. Sibriski , and P. Shvartzmann . 1997. “Awareness of Diagnostic and Clinical Features of Fibromyalgia Among Family Physicians.” Family Practice 14, no. 3: 238–241. 10.1093/fampra/14.3.238. 9201499

[msc70253-bib-0010] Byrne, A. , K. Jones , M. Backhouse , F. Rose , E. Moatt , and C. van der Feltz‐Cornelis . 2023. “Patient and Primary Care Practitioners' Perspectives on Consultations for Fibromyalgia: A Qualitative Evidence Synthesis.” Primary Health Care Research & Development 24: e58. 10.1017/S1463423623000506. 37750736 PMC10540196

[msc70253-bib-0011] Choy, E. , S. Perrot , T. Leon , et al. 2010. “A Patient Survey of the Impact of Fibromyalgia and the Journey to Diagnosis.” BMC Health Services Research 10, no. 102: 1–9. 10.1186/1472-6963-10-102. 20420681 PMC2874550

[msc70253-bib-0012] Colombo, B. , E. Zanella , A. Galazzi , and P. Arcadi . 2025. “The Experience of Stigma in People Affected by Fibromyalgia: A Metasynthesis.” Journal of Advanced Nursing 81, no. 10: 6317–6332. 10.1111/jan.16773. 39835578 PMC12460976

[msc70253-bib-0013] Doebl, S. , G. J. Macfarlane , and R. J. Hollick . 2020. “‘No One Wants to Look After the Fibro Patient’. Understanding Models, and Patient Perspectives, of Care for Fibromyalgia: Reviews of Current Evidence.” Pain 161, no. 8: 716–725. 10.1097/j.pain.0000000000001870. 32701832

[msc70253-bib-0014] Fernandez‐Avila, D. G. , D. N. Rincon Riano , D. M. Ronderos , and J. M. Gutierrez . 2020. “Concepts and Perceptions About the Diagnosis and Treatment of Fibromyalgia in a Group of Colombian Rheumatologists.” Revista Colombiana de Reumatología 27, no. 4: 256–261. 10.1016/j.rcreue.2020.05.003.

[msc70253-bib-0015] Geller, G. , and P. A. Watkins . 2018. “Addressing Medical Students’ Negative Bias Toward Patients With Obesity Through Ethics Education.” AMA Journal of Ethics 20, no. 10: E948–E959. 10.1001/amajethics.2018.948. 30346923

[msc70253-bib-0016] Ghavidel‐Parsa, B. , A. Amir Maafi , Y. Aarabi , et al. 2015. “Correlation of Invalidation With Symptom Severity and Health Status in Fibromyalgia.” Rheumatology 54, no. 3: 482–486. 10.1093/rheumatology/keu355. 25205826

[msc70253-bib-0017] Giusti, E. M. , G. Castelnuovo , and E. Molinari . 2017. “Differences in Multidisciplinary and Interdisciplinary Treatment Programs for Fibromyalgia: A Mapping Review.” Pain Research and Management 2017: 1–19. 10.1155/2017/7261468. PMC5460453 28620267

[msc70253-bib-0018] Hadker, N. , S. Garg , A. B. Chandran , S. M. Crean , M. McNett , and S. L. Silverman . 2011. “Primary Care Physicians’ Perceptions of the Challenges and Barriers in the Timely Diagnosis, Treatment, and Management of Fibromyalgia.” Pain Research and Management 16, no. 6: 440–444. 10.1155/2011/367059. 22184554 PMC3298044

[msc70253-bib-0019] Hahn, S. R. 2001. “Physical Symptoms and Physician‐Experienced Difficulty in the Physician‐Patient Relationship.” Annals of Internal Medicine 134, no. 9: 897–904. 10.7326/0003-4819-134-9_part_2-200105011-00014. 11346326

[msc70253-bib-0020] Harris, P. A. , R. Taylor , B. L. Minor , et al. 2019. “The REDCap Consortium: Building an International Community of Software Partners.” Journal of Biomedical Informatics 95: 1–10. 10.1016/j.jbi.2019.103208. PMC7254481 31078660

[msc70253-bib-0021] Harris, P. A. , R. Taylor , R. Thielke , J. Payne , J. G. Gonzalez , and J. G. Conde . 2009. “Research Electronic Data Capture (REDCap) – A Metadata‐Driven Methodology and Workflow Process for Providing Translational Research Informatics Support.” Journal of Biomedical Informatics 42, no. 3: 377–381. 10.1016/j.jbi.2008.08.010. 18929686 PMC2700030

[msc70253-bib-0022] Häuser, W. , K. Bernardy , B. Arnold , M. Offenbächer , and M. Schiltenwolf . 2009. “Efficacy of a Multicomponent Treatment in Fibromyalgia Syndrome: A Meta‐Analysis of Randomized Controlled Clinical Trials.” Arthritis & Rheumatism 61, no. 2: 216–224. 10.1002/art.24276. 19177530

[msc70253-bib-0023] Hayes, S. M. , G. C. Myhal , J. F. Thornton , et al. 2010. “Fibromyalgia and the Therapeutic Relationship: Where Uncertainty Meets Attitude.” Pain Research and Management 15, no. 6: 385–391. 10.1155/2010/354868. 21165373 PMC3008664

[msc70253-bib-0024] Henry, S. G. , and M. S. Matthias . 2018. “Patient‐Clinician Communication About Pain: A Conceptual Model and Narrative Review.” Pain Medicine 19, no. 11: 2154–2165. 10.1093/pm/pny003. 29401356 PMC6454797

[msc70253-bib-0025] Homma, M. , H. Iskikawa , and T. Kiuchi . 2016. “Association of Physicians’ Illness Perception of Fibromyalgia With Frustration and Resistance to Accepting Patients: A Cross‐Sectional Study.” Clinical Rheumatology 35, no. 4: 1019–1027. 10.1007/s10067-014-2752-6. 25085273

[msc70253-bib-0026] Howman, M. , K. Walters , J. Rosenthal , R. Ajjawi , and M. Buszewicz . 2016. “‘You Kind of Want to Fix It Don’t You?’ Exploring General Practice Trainees’ Experiences of Managing Patients With Medically Unexplained Symptoms.” BMC Medical Education 16, no. 27: 27. 10.1186/s12909-015-0523-y. 26810389 PMC4727318

[msc70253-bib-0027] Karimian, Z. , M. Moradi , N. Zarifsanaiey , and S. Kashefian‐Naeeini . 2024. “Which Educational Messengers Do Medical Students Prefer for Receiving Health Information? Development and Psychometrics of Using Health Messengers Questionnaire.” BMC Public Health 24, no. 139: 1–15. 10.1186/s12889-023-17400-1. 38195427 PMC10777639

[msc70253-bib-0028] Kianmehr, N. , A. Haghighi , A. Bidari , Y. S. Ardekani , and M. A. Karimi . 2017. “Are General Practitioners Well Informed About Fibromyalgia?” International Journal of Rheumatic Diseases 20, no. 12: 1917–1921. 10.1111/1756-185X.12716. 26200844

[msc70253-bib-0029] Kumbhare, D. , S. Ahmed , T. Sander , L. Grosman‐Rimon , and J. Srbely . 2018. “A Survey of Physicians' Knowledge and Adherence to the Diagnostic Criteria for Fibromyalgia.” Pain Medicine 19, no. 6: 1254–1264. 10.1093/pm/pnx271. 29177458

[msc70253-bib-0030] Lawrence, C. , T. Mhlaba , K. A. Stewart , R. Moletsane , B. Gaede , and M. Moshabela . 2018. “The Hidden Curricula of Medical Education: A Scoping Review.” Academic Medicine 93, no. 4: 648–656. 10.1097/ACM.0000000000002004. 29116981 PMC5938158

[msc70253-bib-0031] Mengshoel, A. M. , J. Sim , B. Ahlsen , and S. Madden . 2018. “Diagnostic Experience of Patients With Fibromyalgia – A Meta‐Ethnography.” Chronic Illness 14, no. 3: 194–211. 10.1177/1742395317718035. 28762775

[msc70253-bib-0032] Perrot, S. , E. Choy , D. Petersel , A. Ginovker , and E. Kramer . 2012. “Survey of Physician Experiences and Perceptions About the Diagnosis and Treatment of Fibromyalgia.” BMC Health Services Research 12, no. 356: 356. 10.1186/1472-6963-12-356. 23051101 PMC3502453

[msc70253-bib-0033] Pressimone, C. , C. Cao , J. Kyle , and R. Vanderberg . 2026. “Understanding Essential Aspects of Fibromyalgia Care Through a Qualitative Study of Patient Feedback.” PEC Innovation 8: 100466. 10.1016/j.pecinn.2026.100466. 41858999 PMC12997196

[msc70253-bib-0034] Pressimone, C. , K. Lane , K. Ruppert , et al. 2024. “The Development and Implementation of a Rheumatology Referral‐Based, General Internal Medicine‐Led Fibromyalgia Clinic and Preliminary Patient Outcomes.” Musculoskeletal Care 22, no. 3: e1935. 10.1002/msc.1935. 39261292 PMC11526333

[msc70253-bib-0035] Rice, K. , J. E. Ryu , C. Whitehead , J. Katz , and F. Webster . 2017. “Medical Trainees’ Experiences of Treating People With Chronic Pain: A Lost Opportunity for Medical Education.” Academic Medicine 93, no. 5: 775–780. 10.1097/ACM.0000000000002053. PMC5929494 29140917

[msc70253-bib-0036] Rozenblum, R. , and D. W. Bates . 2013. “Patient‐Centered Healthcare, Social Media and the Internet: The Perfect Storm?” BMJ Quality and Safety 22, no. 3: 183–186. 10.1136/bmjqs-2012-001744. 23378660

[msc70253-bib-0037] Ruben, M. , and N. S. Saks . 2020. “Addressing Implicit Bias in First‐Year Medical Students: A Longitudinal, Multidisciplinary Training Program.” Medical Science Educator 30, no. 4: 1419–1426. 10.1007/s40670-020-01047-3. 34457809 PMC8368581

[msc70253-bib-0038] SAS Institute, Inc . 2023. SAS/STAT 15.3 User’s Guide. SAS Institute, Inc.

[msc70253-bib-0039] Scott, L. , E. Dolan , N. Baker , and Y. Melia . 2023. “Exploring Attitudes of Healthcare Professionals Towards Those With Fibromyalgia: A Q‐Methodological Approach.” British Journal of Pain 17, no. 4: 352–365. 10.1177/20494637231159502. 37538944 PMC10395391

[msc70253-bib-0040] Shattock, L. , H. Williamson , K. Caldwell , K. Anderson , and S. Peters . 2013. “‘They’ve Got Symptoms Without Science’: Medical Trainees’ Acquisition of Negative Attitudes Towards Patients With Medically Unexplained Symptoms.” Patient Education and Counseling 91, no. 2: 249–254. 10.1016/j.pec.2012.12.015. 23369375

[msc70253-bib-0041] Silverwood, V. , C. A. Chew‐Graham , I. Raybould , B. Thomas , and S. Peters . 2017. “‘If It’s a Medical Issue I Would Have Covered It by Now’: Learning About Fibromyalgia Through the Hidden Curriculum: A Qualitative Study.” BMC Medical Education 17, no. 1: 1–9. 10.1186/s12909-017-0972-6. 28899390 PMC5596866

[msc70253-bib-0042] Treufeldt, H. , and C. Burton . 2026. “Stigmatisation in Medical Encounters for Patients With Fibromyalgia: A Focus Group Study.” Patient Education and Counseling 142: 109381. 10.1016/j.pec.2025.109381. 41092545

[msc70253-bib-0043] Vasanthy, B. , and V. C. Parameswaran Nair . 2018. “Fibromyalgia: Perspective of Patients, Medical Students and Professionals.” Journal of Evidenced Based Medicine Healthcare 5, no. 34: 2463–2467. 10.18410/jebmh/2018/508.

[msc70253-bib-0044] Walker, T. , M. Lydston , B. Smedley , and T. Nelson . 2025. Research Suggests Implicit Bias Training Has Positive Impacts on Health Care Workers’ Knowledge, Skills, and Attitudes. Urban Institute. https://www.urban.org/sites/default/files/2025‐05/Research‐Suggests‐Implicit‐Bias‐Training‐Has‐Positive‐Impacts‐on‐Health‐Care‐Workers‐Knowledge‐Skills‐and‐Attitudes.pdf.

[msc70253-bib-0045] Wolfe, F. , D. J. Clauw , M.‐A. Fitzcharles , et al. 2016. “2016 Revisions to the 2010/2011 Fibromyalgia Diagnostic Criteria.” Seminars in Arthritis and Rheumatism 46, no. 3: 319–329. 10.1016/j.semarthrit.2016.08.012. 27916278

