# Supplementary material for: Perceptions of Fibromyalgia Across Career Stages: Survey Results From Internal Medicine Physicians and Medical Students
Source: Musculoskeletal Care. 2026 Aug 3;24(3):e70253. doi: 10.1002/msc.70253 (PMC13433136; doi:10.1002/msc.70253)
Supplement: Supplementary file 1 [file MSC-24-e70253-s001.docx]

Appendix 1. Adapted Difficult Doctor-Patient Relationship Questionnaire-10 (DDPRQ-10)

| Original DDPRQ-10 Question | Adapted DDPRQ-10 Question (All Surveys) | Adapted DDPRQ-10 for Hidden Curriculum Survey (Trainees Only) |
| --- | --- | --- |
| How much are you looking forward to this patient’s next visit after seeing this patient today? | How much are you looking forward to seeing patients with FM*? | How much does your preceptor look forward to seeing patients with FM? |
| How “frustrating” do you find this patient? | How frustrating do you find patients with FM? | How frustrating does your preceptor find patients with FM? |
| How manipulative is this patient? | How manipulative do you find patients with FM? | How manipulative does your preceptor find patients with FM? |
| To what extent are you frustrated by this patient’s vague complaints? | How frustrated are you with FM patients’ vague complaints? | How frustrated is your preceptor by patients with FM’s vague complaints? |
| How self-destructive is this patient? | How self-destructive do you find patients with FM? | How self-destructive does your preceptor find patients with FM? |
| Do you find yourself secretly hoping that this patient will not return? | How much do you find yourself secretly hoping patients with FM will not return? | How much does your preceptor secretly hope patients with FM will not return? |
| How at ease did you feel when you were with this patient today? | How much do you feel at ease with patients with FM? | How at ease does your preceptor feel with patients with FM? |
| How time consuming is caring for this patient? | How time consuming do you find caring for patients with FM? | How time consuming does your preceptor find caring for patients with FM? |
| How enthusiastic do you feel about caring for this patient? | How enthusiastic do you feel about caring for FM patients? | How enthusiastic is your preceptor about caring for patients with FM? |
| How difficult is it to communicate this with patient? | How difficult is it for you to communicate with FM patients? | How difficult is it for your preceptor to communicate with patients with FM? |
|  |  | I feel there is a hidden curriculum that patients with FM are difficult to manage? |

*FM: Fibromyalgia
